# COPI Vesicle Transport Is a Common Requirement for Tube Expansion in *Drosophila*

**DOI:** 10.1371/journal.pone.0001964

**Published:** 2008-04-09

**Authors:** Satish Arcot Jayaram, Kirsten-André Senti, Katarína Tiklová, Vasilios Tsarouhas, Johanna Hemphälä, Christos Samakovlis

**Affiliations:** Department of Developmental Biology, Wenner-Gren Institute, Stockholm University, Stockholm, Sweden; Ecole Normale Superieure, France

## Abstract

**Background:**

Tube expansion defects like stenoses and atresias cause devastating human diseases. Luminal expansion during organogenesis begins to be elucidated in several systems but we still lack a mechanistic view of the process in many organs. The *Drosophila* tracheal respiratory system provides an amenable model to study tube size regulation. In the trachea, COPII anterograde transport of luminal proteins is required for extracellular matrix assembly and the concurrent tube expansion.

**Principal Findings:**

We identified and analyzed *Drosophila* COPI retrograde transport mutants with narrow tracheal tubes. *γCOP* mutants fail to efficiently secrete luminal components and assemble the luminal chitinous matrix during tracheal tube expansion. Likewise, tube extension is defective in salivary glands, where it also coincides with a failure in the luminal deposition and assembly of a distinct, transient intraluminal matrix. *Drosophila* γCOP colocalizes with cis-Golgi markers and in *γCOP* mutant embryos the ER and Golgi structures are severely disrupted. Analysis of *γCOP* and *Sar1* double mutants suggests that bidirectional ER-Golgi traffic maintains the ER and Golgi compartments and is required for secretion and assembly of luminal matrixes during tube expansion.

**Conclusions/Significance:**

Our results demonstrate the function of COPI components in organ morphogenesis and highlight the common role of apical secretion and assembly of transient organotypic matrices in tube expansion. Intraluminal matrices have been detected in the notochord of ascidians and zebrafish *COPI* mutants show defects in notochord expansion. Thus, the programmed deposition and growth of distinct luminal molds may provide distending forces during tube expansion in diverse organs.

## Introduction

The acquisition of optimal tube dimensions during development is critical for the function of organs like the lung, renal and vascular systems. This is reflected by the high incidence of human pathologies associated with tube size defects. For example, cystic overgrowth in the collecting ducts is a landmark of polycystic kidney disease, whereas stenotic tubes cause obstructions of blood vessels and other organs [1], [2]. We use the *Drosophila* respiratory network as a genetic model to understand the determinants of tube size expansion. The tracheal system derives from twenty metameric cell clusters that undergo their last cell division as they invaginate from the epidermis. Cell migration and a series of coordinated tracheal cell rearrangements generate a tubular network that sends branches to all developing tissues [3]–[5]. Branching is followed by three precise morphological events transforming the nascent tubes into mature airways. First, a secretory burst of luminal proteins drives the diametric expansion of the tubes. Later the luminal proteins are cleared and finally, the luminal liquid is replaced by gas to generate a functional respiratory network [6]. Tube diameter expansion occurs within a defined time interval without changes in the number or shape of the constituent cells [7]. During this interval the tracheal epithelium deposits proteins and polysaccharides into the lumen and assembles a massive chitinous extracellular matrix. Mutations affecting chitin biosynthesis (*kkv*) and matrix assembly (*knk*) grow disproportional cystic and over elongated tubes [8]–[10]. In contrast to diametric expansion, tube elongation is continuous during tracheal development. Its termination requires secreted chitin deacetylases that presumably modify the structure of the ECM and thereby provide a stop signal to the epithelium [11], [12]. Collectively, tube size mutants in flies and worms suggest an instructive role of the extracellular luminal matrices in tube growth coordination and termination [13].

What triggers and drives tube expansion? Studies of lumen formation in other systems indicate that apical polarization and targeting of membrane and vesicles to the lumen are central processes in the initiation of tube morphogenesis [2], [14]. In the zebrafish gut, luminal fluid influx generates a distending force that drives coalescence of narrow tubes into a single lumen [15]. The developing trachea tubes and other organs deposit transient solid matrices, which may provide a distending force expanding tube diameter. Mutants affecting COPII vesicle budding and coat assembly show defects in the secretion of luminal proteins and diametric tube growth. The failure in the assembly and expansion of the chitinous matrix may be the underlying cause of the narrow tube phenotypes in these mutants. Alternatively, the diametric expansion defects could be due to reduced delivery of apical membrane or transmembrane regulators to the cell surface [6], [16], [17].

Secreted proteins traffic through the Endoplasmic Reticulum (ER), the Golgi apparatus and the exocytic post-Golgi structures to their final destination. Anterograde and retrograde transport between the ER and the Golgi depends on two distinct types of coated vesicles: COPII vesicles bud from the ER and shuttle nascent secreted proteins to the Golgi apparatus. Conversely, COPI coated vesicles retrieve escaped luminal ER enzymes and recycling cargo adaptor proteins (such as the p23/24 type of proteins) from the Golgi and shuttle them back to the ER [18]. Within the Golgi apparatus, COPI vesicles have been proposed to mediate anterograde transport of secreted cargo or retrograde traffic of Golgi enzymes between different cisterns of the Golgi [19]. The coatomer complex of COPI vesicles is composed of two different layers. The β, δ, γ, ζ subunits form the tetrameric subcomplex of the inner layer. The β- and γ- subunits bind to active Arf1-GTP, γCOP also binds to p23. The outer coat layer subcomplex is trimeric and contains the α, β′ and ε subunits [20].

Here we present the functional analysis of COPI function in tube organogenesis. We find that COPI vesicle transport is required to: a) secrete of multiple luminal proteins, b) assemble a luminal matrix and c) expand the diameter of tubular organs. These defects are not confined to the chitin-lined tracheal tubes but also extend to the salivary glands. In tubular epithelia of *γCOP* mutant embryos, the structure of both the ER and Golgi is compromised indicating that efficient luminal secretion is highly dependant on a functional secretory apparatus. The results highlight the major role of membrane traffic in the expansion of luminal extracellular matrices and apical membrane during tubulogenesis.

## Results

### γCOP is required for tube diameter expansion

**Figure 1:**
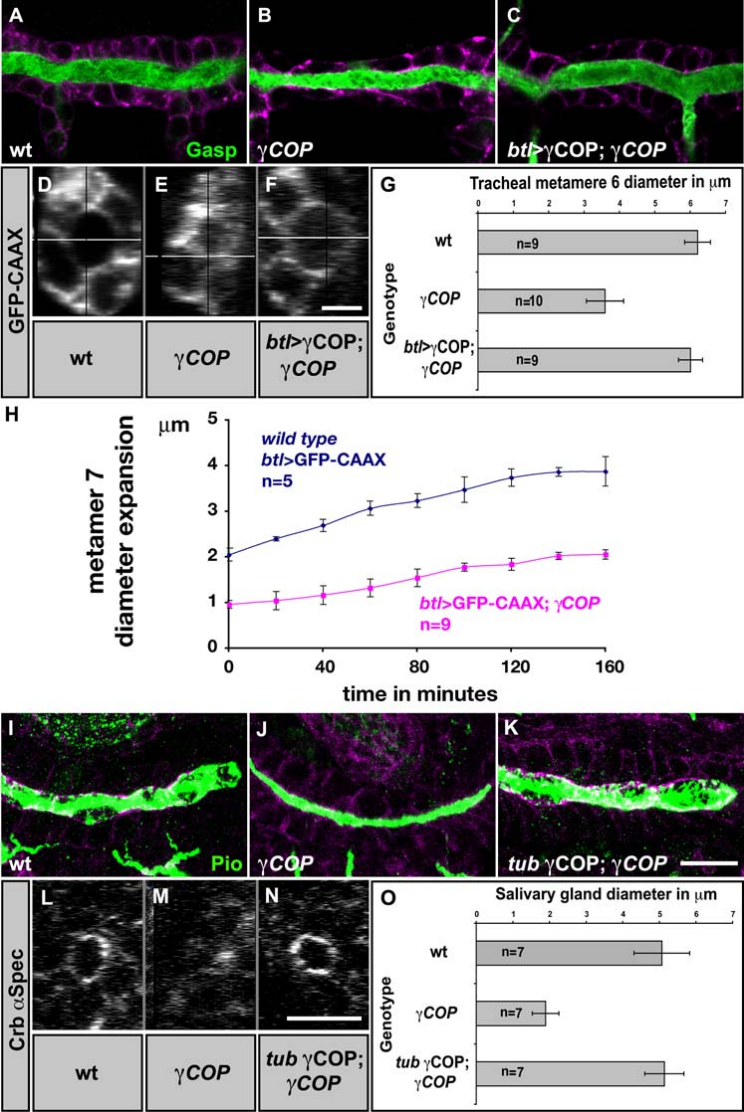
*γCOP* is required for efficient lumen diameter expansion. (A–F, I–N) Confocal micrographs of early stage 16 wild type (A, D, I, L), *γCOP* mutant (B, E, J, M) and *btl*>γCOP; *γCOP*/*γCOP* ^P1^ (C, F) or *tub*-γCOP; *γCOP*/*γCOP* ^P1^ embryos (K, N). GFP stainings of *btl*>GFP-CAAX embryos reveal tracheal DT cells (A–C, in magenta and D–F, in white). Anti-Crumbs and anti-α-Spectrin stainings show SG cells (I–K, in magenta and L–N, in white). Embryos were stained for the luminal antigens Gasp (A–C) and Piopio (I–K) (green). All micrographs show confocal sections except for (D–F) and (L–N) which show yz-confocal sections of DT and SG. (G and O) Lumen diameter of DT metamere 6 using *btl*>GFP-CAAX (G) and diameter of SG measured for Crumbs stainings (O). *γCOP* mutant embryos show significantly (p<0.001) narrower trachea and SG tubes. Transgenic expression of γCOP specifically in the trachea (C, F) or under the *tubulin1α* promoter for SG (K, N) largely rescues the narrow lumen phenotype of *γCOP* mutant embryos. (H) Plot showing DT diameter expansion of live wild type and *γCOP* embryos expressing *btl*>GFP-CAAX. Embryos were recorded from late stage 13 to stage 15. Error bars are means±SD. Scale bars are 10 µm (A–C, I–K), 5 µm (D–F) and 10 µm (L–N).

We identified *γCOP* ^P1^ mutants by screening a collection of lethal P-element insertions for tracheal tube size defects [21]–[25]. At early stage 16, the dorsal trunk (DT) tubes of *γCOP* ^P1^ mutant embryos were narrower compared to wild type. Using imprecise excision, we generated two independent molecular null alleles including the *γCOP* ^Δ114^ lethal allele, which we analyzed for tube expansion (hereafter referred to as *γCOP* mutants) (Figure S1). To visualize the tracheal lumen, we stained embryos for a secreted chitin binding protein, Gasp in embryos expressing GFP-CAAX in the trachea (*btl*>GFP-CAAX) [6]. At early stage 16, the DT tubes of *γCOP* mutants were thinner compared to wild type (Figure 1A, B). This failure in tube expansion was also evident by comparison of yz-confocal sections (Figure 1D, E). Re-expression of *γCOP* in the trachea of mutant embryos rescued the phenotype, indicating that γCOP is required in the tracheal epithelium for diametric tube growth (Figure 1C, F). To quantify tube diameter, we measured the distance between the apical surfaces of cells lining the DT in confocal sections of wild type and *γCOP* mutant embryos expressing *btl*>GFP-CAAX. At early stage 16, the DT diameter in metamere 6 was reduced by 54% in *γCOP* mutants (3.6±0.5 µm, n = 10) compared to wild type (6.2±0.36 µm, n = 9) (Figure 1G). By contrast, DT metamere 6 length was not altered in the mutants (32.7±1.1 µm in wild type n = 4 and 33.1±0.7 µm in *γCOP* mutant embryos n = 4). These measurements indicate that the DT volume capacity of the *γCOP* embryos is reduced to one third compared to wild type.

Wild type embryos initiate diametric tube expansion at stage 14 and nearly complete it within 3.5 hours [6]. To determine when the *γCOP* phenotype is manifested, we imaged live wild type embryos and *γCOP* mutants expressing *btl*>GFP-CAAX. We found that *γCOP* mutant embryos already show a narrower DT diameter at stage 14 and later expand their tubular diameter with a slower rate (0.45 µm/h) compared to wild type (0.71 µm/h) (Figure 1H). Thus, zygotic γCOP function is required before and during the diameter expansion interval.

To investigate where and when *γCOP* is expressed, we performed mRNA *in situ* hybridizations. Maternal *γCOP* transcripts are abundant in early embryos (Figure S1). Zygotic *γCOP* expression commences in the epidermis and salivary glands (SG) from stage 11, initiates in the trachea at stage 13 and at early stage 16 is also detected in the foregut and hindgut tubes (Figure S1). Consistent with the epidermal expression of *γCOP*, we found that *γCOP* mutants fail to complete dorsal closure. This defect can be rescued by ubiquitous *γCOP* expression from a *tubulin1α-*promoter in the mutant background demonstrating that γCOP is required for epidermal morphogenesis (Figure S1).

*γCOP* and other genes encoding components of the secretory apparatus are prominently expressed in developing SGs [26]. This prompted us to analyze SG size growth in *γCOP* mutants. We used antibodies against the apical regulator Crumbs and cytoplasmic α-Spectrin to highlight SG cellular outlines and Piopio to label the SG lumen in wild type and *γCOP* embryos [27]. Like in tracheal tubes, the salivary glands of *γCOP* mutants were thinner compared to wild type (Figure 1I, J, L, M). The SG tube diameter is strikingly reduced in *γCOP* mutants from 5.0±0.76 µm (n = 7) in the wild type to 1.9±0.36 µm (n = 7) in *γCOP* (Figure 1O) (errors are SD). This phenotype can be rescued by ubiquitous expression of *tub1α-γCOP* in mutant embryos (Figure 1K, N, O). Also SG tube elongation is impaired in *γCOP* null mutant embryos. The distance between the most distal cells of the salivary duct expressing *btl*>GFP-CAAX to the gland tip visualized by E-Cadherin (E-cad) staining, was reduced from 50.2±5.8 µm (n = 7) in wild type to 36±4.3 µm (n = 11) in *γCOP* mutants at stage 16 (p<0.001). The similarities of tracheal and salivary gland phenotypes in *γCOP* mutants suggest a common cellular mechanism expanding tubular organs.

### γCOP is required for luminal secretion and assembly of ECM components

**Figure 2:**
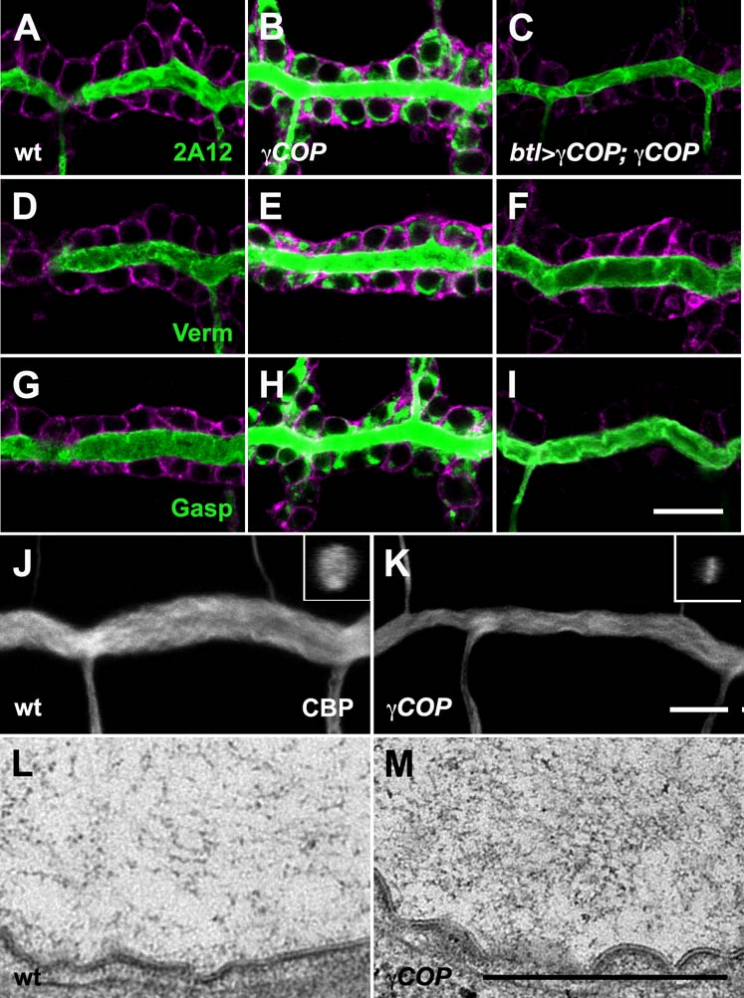
*γCOP* is required for efficient luminal protein secretion and matrix expansion in the trachea. (A–I) Confocal micrographs of DT of wild type (A, D, G, J), *γCOP* mutant (B, E, H, K), *btl*>γCOP; *γCOP*/*γCOP* ^P1^ rescued embryos (C, F, I,). *btl*>GFP-CAAX embryos were stained for the tracheal luminal antigens 2A12 (A–C), Verm (D–F) and Gasp (G–I) (green), GFP (magenta) and with a chitin binding probe (CBP), white in (J,K). All micrographs are single confocal sections except (J–K), which represent confocal projections. The inserts in J and K show y-z projections. *γCOP* mutant embryos show strong intracellular retention of tracheal luminal markers. Transgenic re-expression of *γCOP* largely rescues the luminal secretion phenotype of *γCOP* mutant embryos. (K and M) The luminal ECM is defective in the trachea of *γCOP* mutants. Scale bars are 10 µm in A–K and 0.5 µm in L and M.

A COPII-mediated secretory burst of luminal proteins at late stage 13 drives the diametric expansion of tracheal DT tubes [6]. During this interval luminal chitin and chitin binding proteins assemble into an expanding matrix. To test whether luminal protein deposition is affected in *γCOP* mutants, we stained wild type and mutant embryos expressing *btl*>GFP-CAAX for the luminal antigen 2A12 and the chitin binding proteins Gasp and Verm. While 2A12, Gasp and Verm predominantly localize to the tracheal lumen in wild type stage 15 embryos, those markers are strongly retained inside tracheal cells in *γCOP* mutants (Figure 2A, B, D, E, G, H). This retention can be rescued by tracheal specific *btl*>*γCOP* expression in *γCOP* mutants (Figure 2C, F, I). Thus, γCOP is required in the tracheal epithelium for luminal protein secretion and the completion of diametric tube expansion. Does the defective deposition of chitin binding proteins into the tubes affect the assembly and expansion of the luminal matrix? Staining with a fluorescent chitin binding probe and TEM analysis revealed that the tracheal luminal matrix in *γCOP* embryos remained narrow and dense at early stage 16 embryos (Figure 2J, K, L, M).

**Figure 3:**
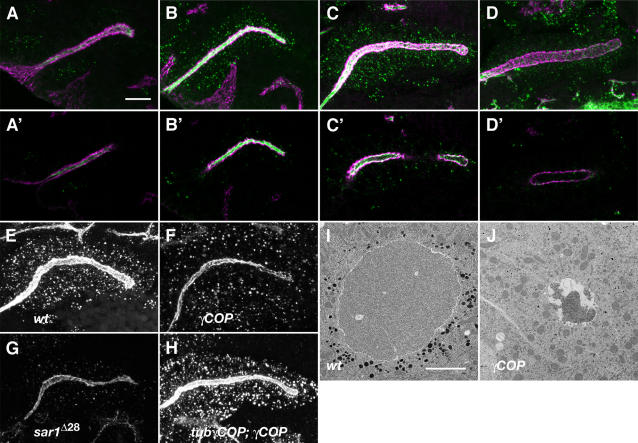
*γCOP* is required for deposition of O-glycans and luminal ECM assembly in the SG. (A–H) Confocal projections show developmental stages of salivary gland (A–D) stained for Tn-antigen (green) and Crumbs in (magenta). (A′–D′) represent confocal sections of the (A–D) projections focused inside the lumen. (A, A′) show stage 11, (B, B′) stage 12, (C, C′) stage 13, (D, D′) stage 15 wild-type embryos. The salivary gland epithelium dynamically deposits Tn antigen into the lumen. While at stage 11 luminal levels of Tn antigen are low, they increase dramatically during stages 12 and 13 and localise to intracellular puncta and lumen. Later, luminal Tn antigen levels decline but a minor proportion remains along the apical epithelial membrane. (E–H) Confocal projections show stage 13 wild-type (E), *γCOP/γCOP* ^P1^ (F), *sar1* ^ΔEP28^ (G) and *tub*-γCOP; *γCOP*/*γCOP* ^P1^ mutant embryos (H) stained for Tn antigen (white). *γCOP* and *sar1* mutant embryos show reduced intracellular puncta and luminal deposition of Tn antigen. (I and J) TEM of salivary gland cross-sections from stage 16 wild-type and *γCOP* mutants. The intraluminal matrix and the electron-dense granules are severely reduced in *γCOP* mutants compared to wild type. Scale bars are 10 µm in A–H. and 2 µm in I and J.

The developing SGs also form an intraluminal matrix. Embryos lacking *pasilla* or a gene cluster encoding putative prolylhydroxylases show disrupted matrix structure and cystic and malformed glands [28], [29]. Do the *γCOP* defects in gland expansion coincide with a failure in the secretion and assembly of the intraluminal matrix? The molecular composition of the SG luminal matrix is unknown, but unlike the tracheal one it does not depend on chitin biosynthesis (data not shown). Recently, the SG lumen has been shown to contain glycans with a single N-acetyl-Galactosamine O-linked to Serine or Threonine [30]. To visualize the luminal secretion of those O-glycans in the salivary gland, we stained fixed embryos with the specific Tn antibody. Directly after SG invagination, minor amounts of Tn antigen were detected in the lumen. The Tn antigen levels markedly increased and localized in intracellular puncta and in the SG lumen at stage 12 and 13. However from stage 14 onwards, the intracellular and luminal levels decline until only very little remains along the apical lining of the SG a stage 15 (Figure 3A, B, C, D). A similar, dynamic localization of Tn antigen is also evident in the trachea (data not shown). Thus, salivary gland expansion is accompanied by a sharp burst of luminal secretion of O-glycosylated proteins. Is *γCOP* required for this secretory burst? Importantly at stage 13, *γCOP* mutant embryos showed strongly reduced intracellular accumulation and luminal deposition of Tn antigens compared to wild type embryos. This phenotype was fully rescued by the *tub1α-γCOP* transgene (Figure 3E, F, H). Like *γCOP*, *sar1* mutant embryos also show a reduced secretion in the SG (Figure 3G), suggesting both COPI and COPII are required for efficient luminal deposition in the SG. To determine if the defects in O-glycan secretion are accompanied by defects in luminal extracellular matrix assembly, we analysed the SGs of *γCOP* mutant embryos by TEM. In striking contrast to wild type embryos that show a uniform and space-filling luminal matrix at early stage 16, *γCOP* mutant embryos showed an atrophic, deformed and abnormally electron dense luminal matrix in the narrow SG lumen (Figure 3I, J). Additionally, the lack of abundant dark apical granules in *γCOP* mutants further supports the role of γCOP in luminal material deposition. Thus, γCOP function is required for the secretion of luminal antigens and assembly of an extracellular matrix.

### *δCOP* mutants phenocopy *γCOP* secretion and diameter phenotypes

**Figure 4:**
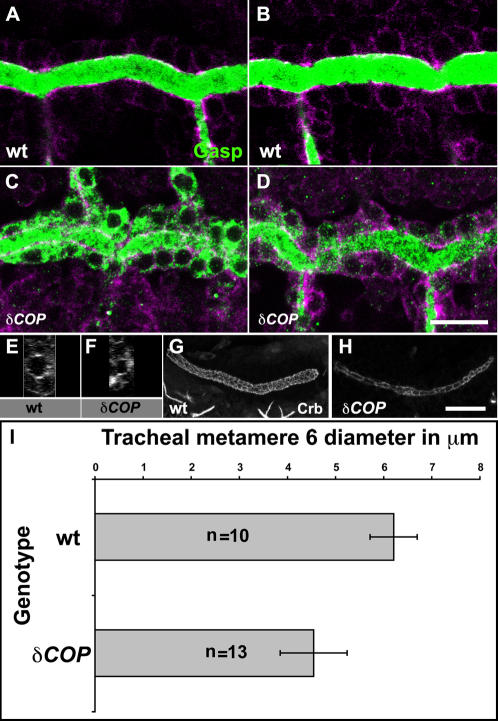
*δCOP* mutants show defects in secretion and luminal diameter expansion. (A–H) Confocal micrographs show the DT in wild type (A, B) and *δCOP* mutants embryos (C, D). Embryos were stained with anti-Gasp (green) together with anti-Crumbs and anti-α-Spectrin to show tracheal cells (magenta). All micrographs are single confocal sections except (E–H) which represent yz- confocal projections of DT of metamere 6 for stage 16 wild type (E), *δCOP* (F) and SG projections of wild type (G), *δCOP* (H). *δCOP* mutant embryos retained a considerable amount of Gasp inside tracheal cells at stage 15 (C). At early stage 16, *δCOP* embryos (D, F) show narrower DT lumen in comparison to wild type (B, E). *δCOP* mutant embryos stained for Crb (H) show narrow SG lumen compared to wild type (G) at stage 16. (I) Graph showing the lumen diameter of the DT at metamer 6 in wild type and *δCOP* embryos at early stage 16 using Crb staining to visualize apical cell membrane. *δCOP* mutant embryos show a significant reduction in lumen diameter (p<0.001) when compared to wild type. Error bars are means±SD. Scale bars are 10 µm.

As the coatomer complex comprises seven subunits that form the COPI vesicle coat, we asked if mutants in other coatomer components show defects in tube expansion. *δCOP* mutants retain the Verm and Gasp luminal proteins inside the tracheal cells at stage 15 and fail to fully expand DT tube diameter at early stage 16 (Figure 4A, B, C, D). The diameter of the DT tube of metamere 6 was reduced by 28% in *δCOP* mutants compared to the wild type (Figure 4E, F, I). A similar phenotype is evident in the SGs (Figure 4G, H). These results confirm that COPI vesicle trafficking mediates luminal secretion and efficient diametric expansion of tracheal and salivary gland tubes.

### Epithelial organization is not affected in zygotic *γCOP* mutants

**Figure 5:**
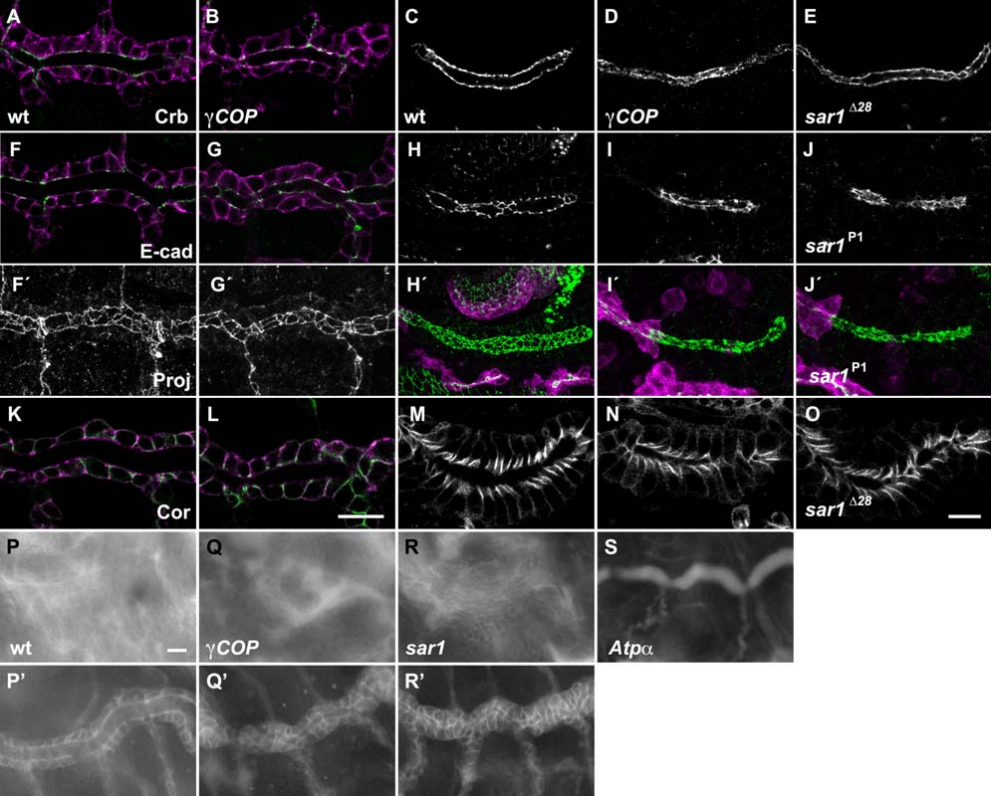
*γCOP* mutant embryos show normal epithelial organization. (A–O) Confocal micrographs show DTs and SG of early stage 16 wild type (A, C, F, F′, H, H′, K, M), *γCOP* mutant embryos (B, D, G, G′, I, I′, L, N) and *sar1* mutant embryos (E, J, J′, O). All micrographs are single confocal sections except (F′–J′) which represent projections. GFP stainings of *btl*>GFP-CAAX embryos label tracheal cells (magenta). The first two columns from the left represent tracheal DT and the next three columns show SG. Embryos were stained for the apical marker Crb (A–E), the adherens junction marker E-cad (F–J) and septate junction marker Coracle (K–O) (either green or white). No defects in the localization or organization of epithelial markers were detected in *γCOP* mutant embryos. (P–S) Wide field images of live wild type (P, P′), *γCOP* (Q, Q′), *sar1^P1^* (R, R′), and *Atpα* (S) embryos injected with 10 kDa fluorescent dextran (P, Q, R, S). The DT is visualized by expression of GFP-CAAX (P′, Q′, R′). The transepithelial barrier function in the trachea is not affected in *γCOP* and *sar1^P1^* mutant embryos. Scale bars are 10 µm.

**Figure 6:**
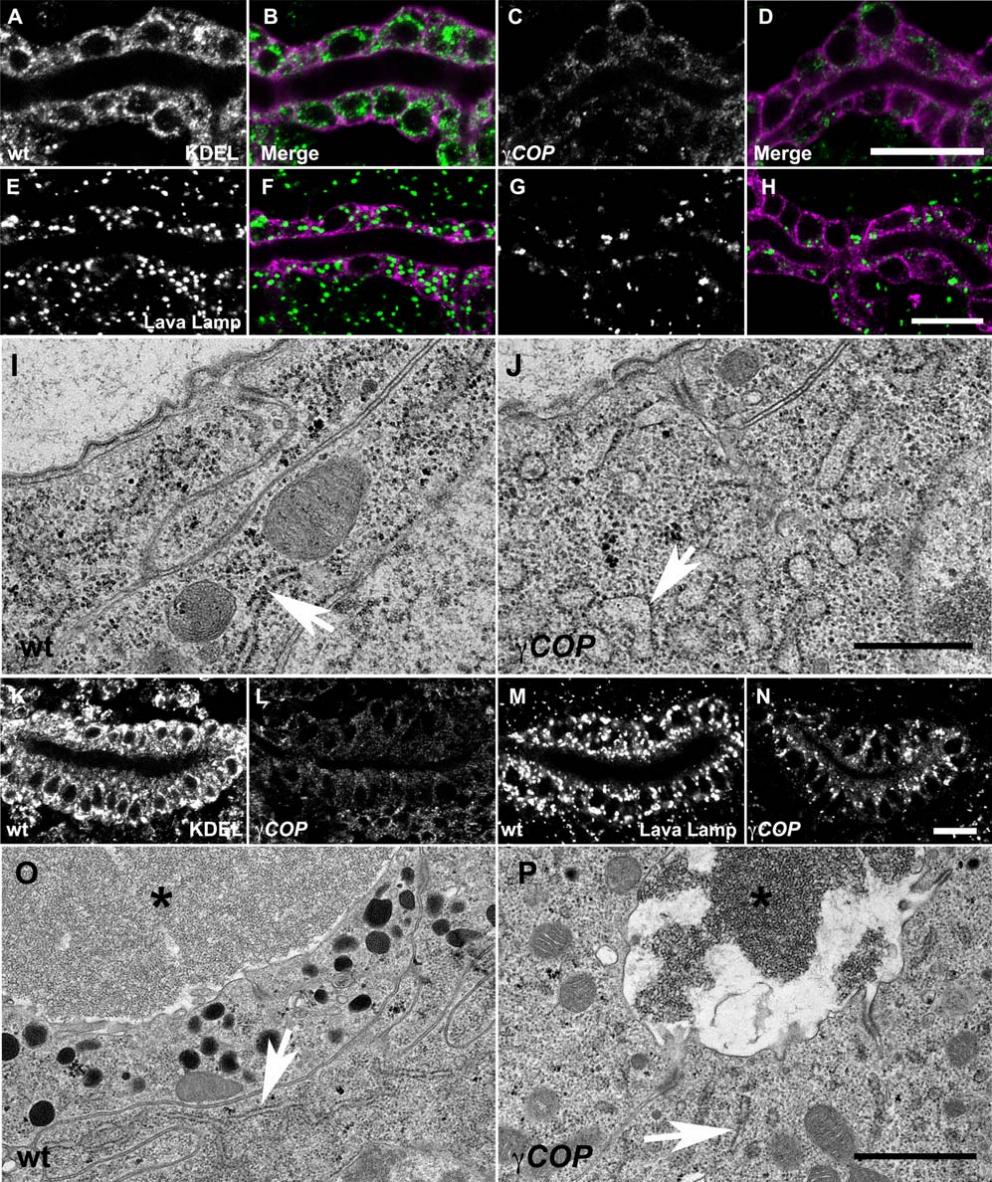
Defective ER and Golgi in *γCOP* embryos. (A–H, K–N) Confocal sections of stage 16 wild type (A, B, E, F, K, M) and *γCOP* mutant embryos (C, D, G, H, L, N) expressing *btl*>GFP-CAAX (magenta). Embryos were stained with anti-KDEL for ER in white (A, C, K, L), green in (B, D) and with anti-Lava lamp for Golgi in white (E, G, M, N), green in (F, H). *γCOP* mutant embryos show reduced ER staining intensity (C, D, L) and a marked decrease in the number of Golgi puncta both in trachea and SG cells (G, H, N). TEM sections of DT (I–J) and SG (O–P) of stage 16 wild type (I, O) and *γCOP* embryos (J, P). In wild type both tracheal and SG cells show a tubular organization of the rough ER, studded with ribosomes (white arrow in I, O). *γCOP* mutant cells show disrupted and bloated rough ER structure (white arrow in J, P). The SG intraluminal matrix is indicated by an asterix in (O) and (P). Scale bars are 10 µm (A–H, K–N), 0.5 µm in (I–J) and 1 µm in (O, P).

Reduced apical secretion in *γCOP* mutants may be an indirect consequence of defects in epithelial polarization. We addressed this by staining for apical and junctional epithelial polarity markers, such as Crumbs, the adherence junction marker E-Cadherin and the septate junction marker Coracle in the trachea and SGs. Except for a minor decrease in the staining intensity of the markers, their localization and the revealed epithelial cell shapes were indistinguishable between mutants and wild type embryos (Figure 5A–O). Further, we found that both adherens junction structure (Figure 6I, J), as well as the transepithelial barrier function of the septate junctions were intact in *γCOP* mutants (Figure 5P–S). Thus, loss of *γCOP* or *sar1* does not affect epithelial integrity (Figure 5E, J, O). In summary, this suggests that apical secretion and luminal matrix assembly are common strategies for tubular organ expansion in *Drosophila*.

### γCOP co-localizes with ER and Golgi markers

We used S2 cells and an antiserum against the mouse γCOP [31] to address the subcellular localization of γCOP in *Drosophila.* The γCOP antibody showed a punctate cytoplasmic signal in S2 cells or cells treated with double stranded (ds) RNA against GFP. *γCOP* dsRNAi treatment abolished the punctate staining and showed a specific reduction of a 96 kDa protein in *γCOP* dsRNAi treated cells compared to GFP dsRNAi treated cells (Figure S2). To reveal the identity of the γCOP puncta, we co-stained S2 cells with either ER or Golgi markers. Confocal analysis showed a partial overlap of γCOP with the ER markers KDEL (the ER retention signal found in many ER proteins) and Calreticulin (Figure S2 and not shown). Co-staining for the cis-Golgi markers GM130 and Lava lamp showed a discrete co-localisation of γCOP with these structures. By contrast, the γCOP puncta did not overlap with the median (gp120) and trans Golgi marker (peanut agglutinin) staining (Figure S2). Thus γCOP localizes in the ER and cis-Golgi units in *Drosophila* cells, consistent with the localization of its mammalian and plant homologs [31], [32].

### ER and Golgi are disrupted in *γCOP* mutant embryos

The prominent secretory defects in the *γCOP* mutants and the co-localization of γCOP with ER and cis-Golgi markers suggested that the basal secretory apparatus may be defective in *γCOP* embryos. We therefore stained wild type and mutant embryos for KDEL, the transmembrane adaptor p23/Baiser and Calreticulin, an abundant ER chaperone [33]–[35]. We observed a drastic reduction in the staining intensities of all markers in the trachea and SGs of the mutants compared to wild type embryos (Figures 6A–D, K, L and S3) suggesting a disruption of ER integrity in *γCOP* embryos. The reduced intensity of Calreticulin staining in *γCOP* mutants was also evident in epidermal cells (Figure S3), which also showed an abnormally high intracellular accumulation of Verm at stage 16 (data not shown). We used TEM to visualize the tracheal and SG ER in wild type and *γCOP* mutants. In contrast to the uniformly organized tubular ER of the wild type, ER structures were severely bloated in the mutants particularly in the trachea (Figure 6I, J, O, P). These structural defects are accompanied by a mild activation of the unfolded protein response evidenced by the presence of processed *xbp1* transcripts in *γCOP* mutants (Figure S3) [36]. The above data show that COPI vesicle transport is required for maintenance of ER integrity in epithelial tissues. We analyzed the Golgi status by staining embryos for the Lava lamp and gp120 markers [37]. As expected the intensity of Lava lamp and gp120 puncta were reduced in the trachea (Figure 6E–H) and salivary glands of mutant embryos (Figures 6M, N and S3). Thus, the deficits in luminal protein deposition and tube expansion are due to structural defects in the secretory apparatus of *γCOP* mutants.

### COPI and COPII vesicle trafficking drive tube expansion

**Figure 7:**
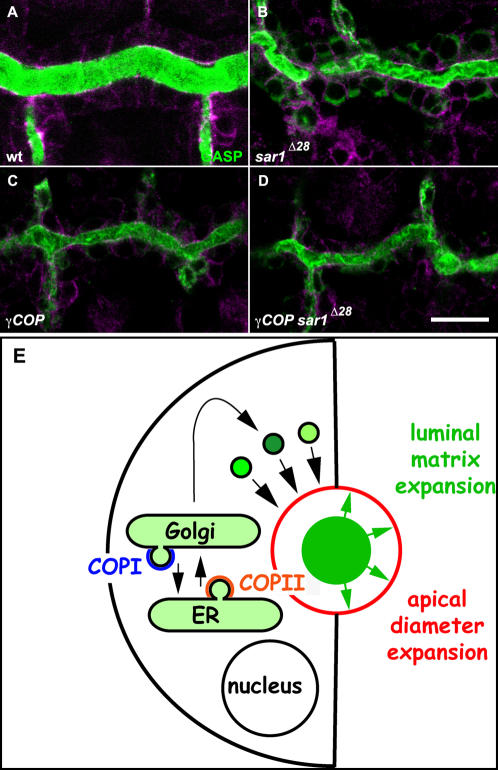
COPI and COPII function in tube size control. (A–D) Confocal sections of early stage 16 wild type (A), *sar1* ^Δ28^ (B), *γCOP* (C) and *sar1* ^Δ28^ *γCOP* double mutant embryos (D). Cells of tracheal DT stained for anti-Crumbs and anti-α-Spectrin shown in magenta (A–D) and for the tracheal luminal antigen Gasp (A–D) (green). (E) Schematic illustration of a cross-section through an epithelial tube. The ER, Golgi and post-Golgi vesicles carry secreted proteins (green) into the lumen. Both COPI and COPII vesicular transport between the ER and Golgi are required for intraluminal matrix assembly and apical membrane addition (red). Green arrows indicate the proposed pressure exerted on the cells by the expanding matrix. Scale bars are 10 µm.

COPI coatomer subunit mutations cause tube expansion and cellular defects that closely resemble the phenotypes of COPII coat mutants [6]. However, only *γCOP* mutants show dorsal closure phenotypes suggesting a selective role of COPI vesicles. To determine if mutants in COPI and COPII components show additive phenotypes in the trachea, we analyzed zygotic single *sar1* or *γCOP* null mutant embryos and *sar1 γ*COP double mutants. Interestingly, *sar1 γCOP* double mutant embryos showed as narrow DT tubes, as *sar1* or *γCOP* single mutants (Figure 7B, C, D). The lack of an additive phenotype in the double mutants suggests that anterograde COPII and retrograde COPI vesicles transport are equally important to maintain both ER and Golgi structures and that the dynamic bidirectional ER-Golgi traffic is essential for the secretory activity of epithelial cells during tube expansion.

## Discussion

COPI and COPII mutations strictly affect diameter but not length expansion in the trachea. SG expansion is mainly defective in diameter and to lesser degree also in length. This differential effect may be explained by the dramatic expansion of SG in both diameter and length during the SG secretory burst. The tracheal tubes on the other hand, elongate continuously and the programmed boost of secretory activity precedes the short interval of diametric expansion.

*γCOP* and *sar1* mutants show qualitatively similar phenotypes in tube expansion in the trachea and the SG. *γCOP* and *sar1* gene products are deposited in the oocyte at sufficient levels to support early embryogenesis. Distinct levels in maternal contribution or zygotic expression of γCOP and Sar1 may contribute to the quantitative differences in tube expansion phenotypes. In sharp contrast to the similarities of tubular defects, *γCOP* mutant embryos fail to complete dorsal closure while *sar1* mutants close normally (data not shown). While we cannot exclude that the γCOP maternal product is less stable than Sar1, this phenotypic difference suggests that not all developmental processes require both COPII and COPI function equally. This may be attributed to a specific COPI function in intra Golgi trafficking.

The developing trachea and SG tubes initiate a dynamic secretory burst that deposits transient solid matrices. The diametric expansion defects in mutants for COPI and COPII components may in part be due to reduced delivery of apical membrane or transmembrane regulators to the cell surface. The strict correlation between the luminal matrix assembly defects and the tube expansion phenotypes strongly suggest that the swelling luminal matrixes directly enlarge the tubes from the inside (Figure 7E). This is analogous to the distending pressure from the transient fluid influx into the lumen that expands the lung and coalesces the gut lumen in zebrafish [15], [38]. “Inside out molding” by swelling luminal matrixes may be a common mechanism in tubulogenesis extending to vertebrates. The notochord of many ascidian species expands by coalescing vacuoles into a single continuous lumen stretching through the entire notochord [39], while other vertebrate species rely on an immense intracellular vacuolarisation [40]. The luminal/vacuolar turgor of the notochord is contained by a thick basement membrane ECM. This renders the expanding notochord into a stiff, rod-shaped organ that elongates the tail and the entire animal [41]. The luminal secretion of water absorbing glycosaminoglycans has been proposed to generate the turgor for notochord expansion [42]. Zebrafish mutants in coatomer encoding genes fail to expand their notochord and larval tail. These phenotypes could be partly due to defects in laminin secretion and the assembly of the basal extracellular matrix [43] . The recent visualisation of vacuolar membrane fusion with the luminal cavity in ascidians and our analysis of *COPI* mutants further implicate luminal secretion in notochord expansion. Thus, the programmed deposition of swelling luminal matrices may be a common strategy of tube expansion.

## Materials and Methods

### *Drosophila* Strains

The P element alleles *l(3)s057302* and *KG06383* (*γCOP* ^P1^ and *γCOP* ^P2^ respectively; Figure S1) fail to complement each other. Insertion sites were determined by plasmid rescue and PCR. P-element excision of *γCOP* ^P2^ generated a precise excision reverting the tracheal phenotype, along with two independent null lethal alleles: *Δ128,* a smaller deletion, taking out the 5′UTR and a major part of the first exon and *Δ114* (*γCOP* in text) which lacks the 5′UTR, first exon and the majority of second exon (Figure S1). All the analysis was performed on *Δ114*. Rescue with *btl*>γCOP or *tubulin1α*-γCOP was performed in *γCOP*/*γCOP* ^P1^ trans-heterozygous mutant background. Other mutants used were *KG07426* (*δCOP*), *l(3)05712* (*sar1* ^P1^), *sar1* ^Δ28^ (*sar1* ^EP3575Δ28^) [6], *kkv* ^DZ8^ [8], *Atpα* ^S067611^ [22], and *NP5464* (*p23/baiser*).

### Molecular biology

#### ds-RNAi, RT-PCR

pUAST-γCOP and *tubulin1α*-γCOP were generated by sub-cloning the cDNA from RE37840 into pUAST and p*tub1alpha*-GAL80 [44]. Down regulation of *γCOP* by RNAi in S2 cells was performed as described in [45]. Primer pairs tailed with T7 RNA polymerase promoter were used to amplify a PCR fragment from the cDNA clone. Primers used in PCR amplification 5′-TTAATACGACTCACTATAGGGAGACCAGGAGGCTTTGAACAGCGACA-3′ and 5′-TTAATACGACTCACTATAGGGAGACGCACAATAGGGCTCTCCAAGATGA-3′. The 900 bp PCR product was then used as template for dsRNA production with the MEGAscript RNAi kit (Ambion).

For RT-PCR, total mRNA was isolated from stage 16 wild type and *γCOP* mutant embryos using oligo(dT)-coupled beads (Dynabeads). Unfolded protein response was induced in S2 cells by treatment with 10mM DTT. Reverse transcription was performed with SuperScript-II (Invitrogen). Primers flanking the splice-site of *xbp-1* mRNA were used, 5′-CGCCAGCGCAGGCGCTGAGG-3′ and 5′-CTGCTCCGCCAGCAGACGCGC-3′. *actin* mRNA was amplified using 5′-GACCCAGATCATGTTCGAGACC-3′ and 5′-GCATTTGCGGTGAACGATTCCGGG-3′ on the same beads to provide a quantification control.

### Immunostaining, TEM and Western blotting

Immunostainings, Western blots and TEM were performed as in [6], [45] with the following additional antibodies: Rabbit anti-Mouse γCOP1 raised against C-terminal region [31] (The *Drosophila* γCOP protein is 50% identical to mouse γCOP1), rabbit anti-Lava Lamp [37]. rabbit anti-*Galleria mellonella* calreticulin [35]. Rabbit anti-p23/Baiser raised against cytoplasmic peptide tail [33]. Fluorescein-Peanut agglutinin (PNA) (Sigma), rabbit anti-dGM130 [46] (Abcam). The mAB anti-Tn clone B1.1 (Biomeda) [30] recognizes the unmodified N-acetyl-Galactosamine-group O-linked to serines or threonines on protein substrates (Tn antigen). To examine the SJ barrier function in tracheal cells, embryos were injected at late stage 16 with 10 kDa Rhodamine-Dextran (Molecular Probes).

## Supporting Information

Figure S1 *γCOP* genomic locus and expression pattern. (A) The *γCOP* locus and positions of P element insertions and deletions. (B–E) Bright field images of wild type stage 1 (B), early stage 16 (C) and *γCOP* mutant embryos stained for *γCOP* (E) with anti-sense RNA probes (grey). (D) shows an embryo stained with a “sense” RNA probe. Expression of *γCOP* transcripts was strongly reduced in zygotic *γCOP* mutant (E). (F–K) Confocal sections of *1-eve-1* embryos showing zygotic expression of *γCOP* in the SG (F) and DT (H, I) with *γCOP* anti-sense probes. No staining was detected with the sense probe in SG (G) and DT (J, K). Tracheal cells are visualized by anti-β-Gal staining (magenta in I, K). Zygotic expression of *γCOP* transcripts is observed in SG and trachea. (L–N) Confocal projections of wild type (L), *γCOP* mutant (M) and *tub*-γCOP; *γCOP/γCOP* ^P1^ embryos (N) stained for Coracle to visualize the dorsal epidermis. *γCOP* mutant embryos fail to close dorsally. Scale bars are 30 µm in (B–E) and 10 µm in (F–G, H–K, L–N).
(4.14 MB TIF)

Figure S2 γCOP co-localizes with ER and Golgi markers. (A–J) Confocal sections of S2 cells stained with anti-γCOP (green) and gp120 (A–D, I), or KDEL (F), or GM130 (G), or Lava lamp (H), or PNA (J) (red). S2 cells were either mock treated (A) or treated with dsRNA for GFP (B) or for *γCOP* for 3 days (C) or 6 days (D). Golgi and γCOP staining were reduced in *γCOPi* treated cells. (E) Western blot of S2 cell extracts show a marked reduction of ∼97 kDa protein (equivalent to predicted molecular weight) in ds*γCOP* treated cells, but not in untreated cells. Lamin was used as loading control. γCOP shows partial overlap with KDEL and clear co-localization with cis-Golgi markers. Scale bars are 10 µm.
(4.12 MB TIF)

Figure S3 Defective ER and Golgi in *γCOP* embryos. (A–N) Confocal sections of wild type (A, B, E, F, I, M), *γCOP* mutant embryos (C, D, G, H, J, N), heterozygous for *p23/baiser* (K) and *P23/baiser* mutant embryos at early stage 16 (L). (A–H) Embryos expressing *btl*>GFP-CAAX were stained for the ER marker Calreticulin for ER in white (A, C), green in (B, D) and the Golgi markers gp120 for Golgi in white (E, G), or green (F, H). GFP localization in the DT is in magenta. (I–L) show SG staining for p23/Baiser and (M, N) depict epidermis stained for Calreticulin. *γCOP* mutants show reduced ER staining intensity (C, J, N) and a decreased number of Golgi units (G). (O) An agarose gel showing RT-PCR products detecting splicing of *XbpI* mRNA in *γCOP* mutant embryos. *γCOP* mutants show a mild Unfolded Protein Response (UPR). Scale bars are10 µm.
(5.06 MB TIF)
