# Supplementary figures and images for: COPI Vesicle Transport Is a Common Requirement for Tube Expansion in Drosophila
Source: PLoS One. 2008 Apr 9;3(4):e1964. doi: 10.1371/journal.pone.0001964 (PMC2276865; doi:10.1371/journal.pone.0001964)

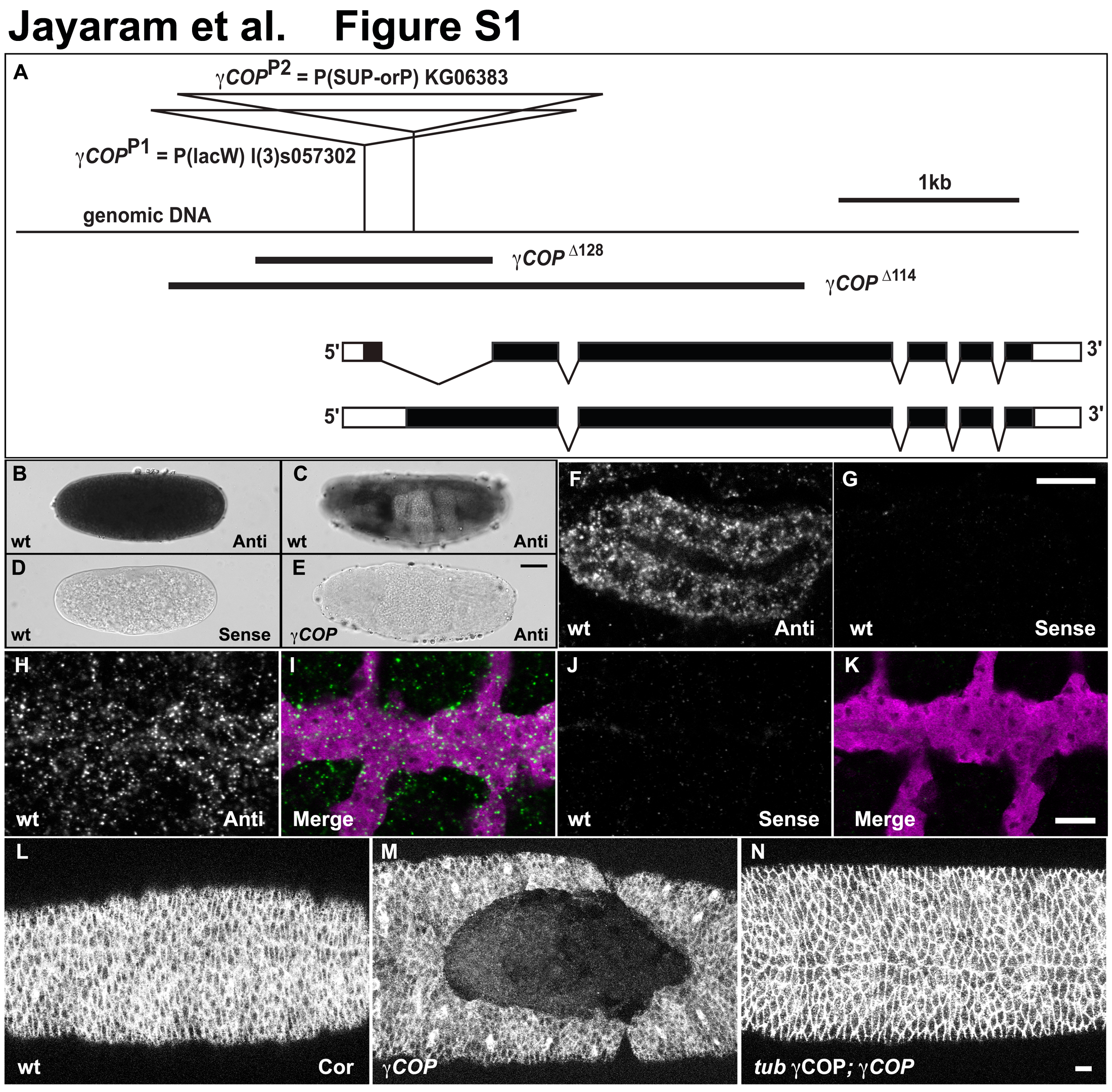

Supplement: Figure S1 — γCOP genomic locus and expression pattern. (A) The γCOP locus and positions of P element insertions and deletions. (B–E) Bright field images of wild type stage 1 (B), early stage 16 (C) and γCOP mutant embryos stained for γCOP (E) with anti-sense RNA probes (grey). (D) shows an embryo stained with a “sense” RNA probe. Expression of γCOP transcripts was strongly reduced in zygotic γCOP mutant (E). (F–K) Confocal sections of 1-eve-1 embryos showing zygotic expression of γCOP in the SG (F) and DT (H, I) with γCOP anti-sense probes. No staining was detected with the sense probe in SG (G) and DT (J, K). Tracheal cells are visualized by anti-β-Gal staining (magenta in I, K). Zygotic expression of γCOP transcripts is observed in SG and trachea. (L–N) Confocal projections of wild type (L), γCOP mutant (M) and tub-γCOP; γCOP/γCOP P1 embryos (N) stained for Coracle to visualize the dorsal epidermis. γCOP mutant embryos fail to close dorsally. Scale bars are 30 µm in (B–E) and 10 µm in (F–G, H–K, L–N). (4.14 MB TIF) [file pone.0001964.s001.tif]

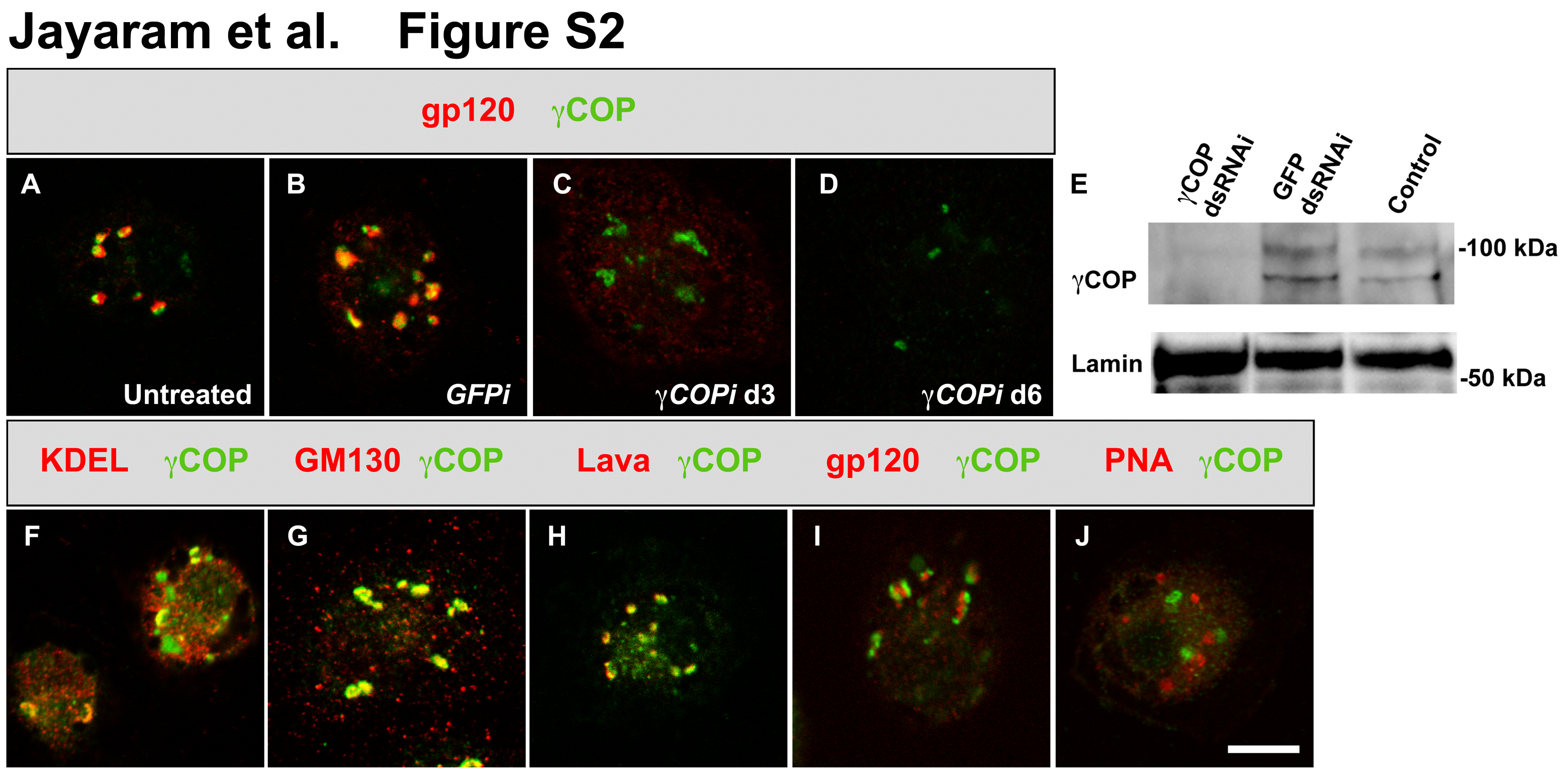

Supplement: Figure S2 — γCOP co-localizes with ER and Golgi markers. (A–J) Confocal sections of S2 cells stained with anti-γCOP (green) and gp120 (A–D, I), or KDEL (F), or GM130 (G), or Lava lamp (H), or PNA (J) (red). S2 cells were either mock treated (A) or treated with dsRNA for GFP (B) or for γCOP for 3 days (C) or 6 days (D). Golgi and γCOP staining were reduced in γCOPi treated cells. (E) Western blot of S2 cell extracts show a marked reduction of ∼97 kDa protein (equivalent to predicted molecular weight) in dsγCOP treated cells, but not in untreated cells. Lamin was used as loading control. γCOP shows partial overlap with KDEL and clear co-localization with cis-Golgi markers. Scale bars are 10 µm. (4.12 MB TIF) [file pone.0001964.s002.tif]

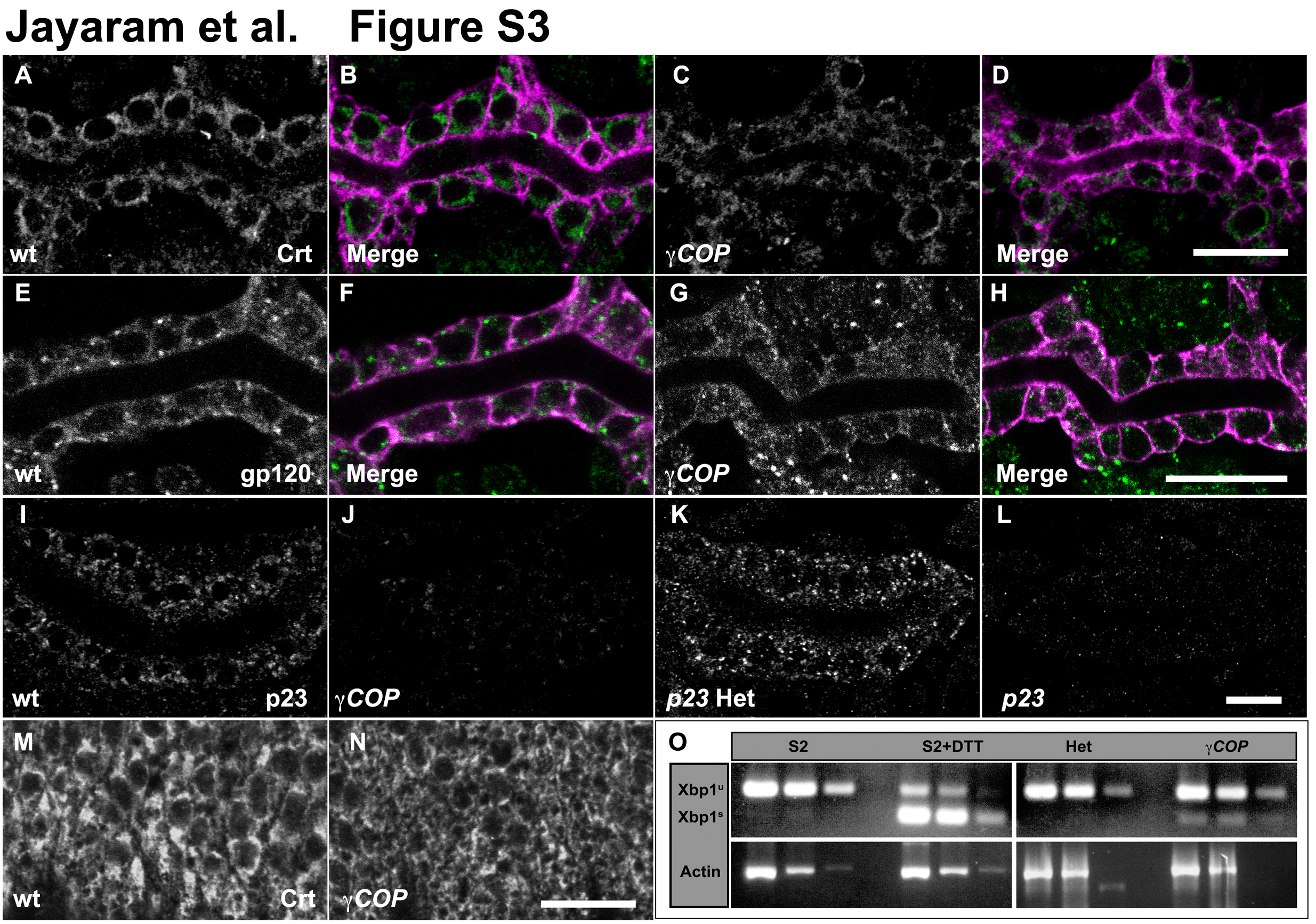

Supplement: Figure S3 — Defective ER and Golgi in γCOP embryos. (A–N) Confocal sections of wild type (A, B, E, F, I, M), γCOP mutant embryos (C, D, G, H, J, N), heterozygous for p23/baiser (K) and P23/baiser mutant embryos at early stage 16 (L). (A–H) Embryos expressing btl>GFP-CAAX were stained for the ER marker Calreticulin for ER in white (A, C), green in (B, D) and the Golgi markers gp120 for Golgi in white (E, G), or green (F, H). GFP localization in the DT is in magenta. (I–L) show SG staining for p23/Baiser and (M, N) depict epidermis stained for Calreticulin. γCOP mutants show reduced ER staining intensity (C, J, N) and a decreased number of Golgi units (G). (O) An agarose gel showing RT-PCR products detecting splicing of XbpI mRNA in γCOP mutant embryos. γCOP mutants show a mild Unfolded Protein Response (UPR). Scale bars are10 µm. (5.06 MB TIF) [file pone.0001964.s003.tif]
